# Ambulance Arrangements

**Published:** 1900-09-15

**Authors:** 


### AMBULANCE ARRANGEMENTS.

A good deal of attention was given at the Ipswich meeting of the British Medical Association to questions bearing upon the transport of the sick, and the giving of first aid to the wounded, both in war and in civil life. Among other papers was an interesting one by Surgeon-Major Hutton, of the St. John Ambulance Association, giving the history of the measures which have been taken with this object from time to time during recent years. He concludes that the proper authority to carry out an efficient ambulance service in large towns is the municipal authority through the police force, the police being on duty day and night, and therefore available at all times and hours. He recommends, therefore, that in large towns the ambulance service should be controlled by the municipalities, the police being required to pass through the course of instruction and hold the certificate of the St. John Ambulance Association, and also that they should be re-examined annually by a medical officer appointed by the Association. In smaller towns, where there are local boards, he recommends that these bodies should control the ambulance service, getting assistance as they may require it from the members of the St. John Ambulance Brigade when such a body exists in the locality; while in country villages it is desirable that a St. John stretcher should be kept for use in the village police-station, and in large villages a wheeled litter also. It is also desirable, he says, that the St. John Ambulance instruction should be carried, by means of classes in first-aid, into all villages, so that ready and practical assistance may be at hand in case of accident or for the removal of the sick and injured. Surgeon-Major Hutton also drew special attention to the Invalid Transport Corps, established mainly by the advice of Sir John Furley, at St. John's Gate, Clerkenwell. This corps was, he said, prepared to move invalids on payment to any part of the United Kingdom, and also abroad. He felt sure that its services might often be found of immense assistance, especially in difficult cases. Dr. John J. de Zouche Marshall sketched out a very complete scheme for the spread of knowledge of ambulance work throughout the country. His plan has, at least, the advantage of thoroughness. He recognises that the labourer is worthy of his hire, and that in many positions a man with a good working knowledge of first-aid is a more valuable public servant than one who is ignorant on such matters, and he very logically urges that policemen and other public servants who take the trouble to render themselves "professional ambulancers" should receive special extra pay. But, in addition to such public officers, he would have intelligent men of good physique all over the country enrolled and trained under the banner of the St. John Ambulance Brigade, so that every village might have its squad and every town its company. He would, indeed, have the St. John Ambulance Brigade take the entire ambulance protection of the people into its own hands, and have them organised and paid accordingly. It is not quite clear, however, by whom all these intelligent men of good physique are to be paid, nor whence is to come the money to pay fees to the medical instructors.

